# Residential transience among US adolescents: association with depression and mental health treatment

**DOI:** 10.1017/S2045796018000823

**Published:** 2019-01-15

**Authors:** C. Glasheen, V. Forman-Hoffman, S. Hedden, T. Ridenour, J. Wang, J. Porter

**Affiliations:** RTI International, Behavioral Health Research Division, Research Triangle Park, NC 27709, Unites States of America

**Keywords:** Depression, housing instability, mental health services, residential mobility

## Abstract

**Aims:**

Residential instability, including transience (i.e. unusually frequent mobility), is associated with higher risk for emotional and behavioural problems in children and young adults. However, most studies have not compared the effect of recent *v.* more distal moves on mental health or on mental health treatment. This study examined associations between recent (past year) and distal (past 2–4 years) residential transience and past year major depressive episode (MDE) and mental health treatment in a nationally representative sample of US adolescents aged 12–17.

**Methods:**

Data are from the 2010–2014 National Surveys on Drug Use and Health (*n* = ~107 300 adolescents). *T*-tests were used to examine the prevalence of MDE by number of moves in the past 5 years among a nationally representative sample of adolescents. Additionally, multivariable logistic regression models were used to evaluate the adjusted association between recent (⩾2 moves in the past year) and distal (⩾4 moves in the past 5 years, but no recent transience) and (1) past year MDE and (2) past year mental health treatment among adolescents with MDE.

**Results:**

MDE prevalence increased linearly with number of moves in the past 5 years (*p* < 0.001). The adjusted odds of MDE were greater among youths with distal transience (adjusted odds ratio (AOR) = 1.25, 95% confidence interval (CI) = 1.09–1.44) and among those with proximal transience (AOR = 1.31, 95% CI = 1.17–1.46), compared with those without transience in the past 5 years. The MDE prevalence did not differ between those with distal and proximal transience (*p* = 0.163). In youths with past year MDE, the prevalence of past year mental health treatment was greater among those with proximal transience compared with those without transience (AOR = 1.40, 95% CI = 1.15–1.70), but there was no significant difference in treatment among those with distal *v.* no transience.

**Conclusions:**

Distal and recent transience are associated with past year MDE among adolescents. Adolescents with MDE who had recent transience were more likely to receive past year mental health treatment compared with those without transience. However, those with only distal transience were not more likely to receive treatment. Parents, school officials and health care providers should be aware that residential mobility in the past 5 years may indicate increased odds of depression among adolescents even among adolescents whose housing stability has improved in the past year.

## Introduction

The deleterious effects of depression are staggering – depression is the tenth leading cause of disability-adjusted life years lost (McKenna *et al*., 2005) and an attributable risk for about 50% of all suicidal ideation (Goldney *et al*., 2000). Identifying risk factors for depression for targeted interventions may prevent or reduce the consequences of depression. One particular risk factor for depression that has received a fair amount of research is residential mobility and residential transience.

Residential transience is loosely defined as frequent residential mobility (Breakey and Fischer, 1995; Clark, 2010). This can be a stressful life event that, depending on frequency, may become a chronic stressor (Young and Dietrich, 2015). Several studies have demonstrated the association between residential instability and emotional and behavioural problems in children and young adults (Simpson and Fowler, 1994; Gilman *et al*., 2003; Qin *et al*., 2009). It is hypothesised that mobility can disrupt existing family roles and routines, create competing resource demands on parents and children (e.g. time, energy, attention), and disrupt social networks (Anderson *et al*., 2014). As a result, adolescents may eventually give up on forming attachments with peers and teachers at school if they anticipate frequent mobility. Among adolescents in particular, social support networks, especially peer support networks (Hostinar *et al*., 2015), are important buffers to life stress (Hostinar and Gunnar, 2015; Tennant *et al*., 2015). In addition to the stress created by frequent moving being a risk factor for mental health problems (Conway *et al*., 2016), disruption in social support networks is as well. Studies have indicated that moving to a neighbourhood with better socioeconomic conditions during adolescence may have at least short-term deleterious mental health effects, possibly due to stress and disruption of social networks (Kessler *et al*., 2014; Chetty *et al*., 2016).

Several studies have documented an association between residential transience and depression. Most of these studies, however, have had small sample sizes that targeted special populations, such as military families (Kelley *et al*., 2003), regional samples of racial/ethnic minorities (DeWit, 1998), inpatients (Mundy *et al*., 1989), low-income families in a housing intervention trial (Kessler *et al*., 2014), or children in Head Start programmes (Stoneman *et al*., 1999). Although the findings of these studies have been largely consistent, the use of specialised samples make the results non-generalisable to more diverse populations.

The few, larger and more generalisable studies that exist have shown an association with transience and mental health. For instance, a regional sample of over 1000 children participating in the National Collaborative Perinatal Project found that moving three or more times before age 7 was associated with 36% greater likelihood of lifetime major depression and more than twice the likelihood of developing depression before age 14 compared with those who moved less (Gilman *et al*., 2003). However, the high sample attrition (47%) leads to concerns over the reliability of the results. More recently, analyses of the National Survey on Drug Use and Health demonstrated that just one move in the past 5 years was associated with increased past year MDE among adolescents (Susukida *et al*., 2015), and a national study in Denmark found an increased risk for a full spectrum of mental illnesses associated with cross-municipality residential moves during childhood and adolescents (Mok *et al*., 2016).

Residential transience may also impede access to mental health services, although few studies have investigated this topic. In a 2008 systematic review of the literature, only five studies were identified that examined any type of residential instability and the association with health care utilisation in childhood and adolescent. Three of the studies found decreased routine and/or preventative health care utilisation among those with mobility. Despite the potential for residential transience to disrupt healthcare access, continuity of care and trusted therapeutic relationships, no studies were identified that had examined mental health care, specifically (Jelleyman and Spencer, 2008). Frequent moves that require repeated establishment of relationships with new providers because of residential distance or changes to health insurance coverage may make it difficult to establish patient–practitioner rapport and eventually discourage treatment seeking (Lindsey *et al*., 2014). Moreover, relocation often requires the use of significant parental resources such as time, energy and money, potentially reducing the ability for parents to seek mental health treatment for their children or adolescents (Anderson *et al*., 2014).

The purpose of this investigation is to examine the relationship between recent (past year) and distal (past 5 year) residential transience and major depressive episodes (MDE) using a nationally representative sample of US adolescents aged 12–17 and to test hypotheses driven by prior research that suggests an association between transience and reduced continuity of health care among adolescents with MDE (Jelleyman and Spencer, 2008). Three hypotheses guided these analyses:

• Adolescents with residential transience will be more likely to have past year MDE than those without.
• Adolescents with recent (past year) residential transience will be more likely to have past year MDE than those with distal (>1 year ago) transience.
• Among adolescents with MDE, those with residential transience will be less likely to report past year receipt of mental health services than those without transience.

## Method

### Participants

Data are from the 2010 to 2014 NSDUHs, a nationally representative cross-sectional annual survey of approximately 68 500 people aged 12 or older in the USA, sponsored by the Substance Abuse and Mental Health Services Administration. NSDUH uses a stratified multistage area probability sample of residents of households and non-institutional group quarters (e.g. shelters, boarding houses, halfway houses) to produce a nationally representative sample of the US civilian non-institutionalised population (Center for Behavioral Health Statistics and Quality, 2015*b*). Individuals in selected households are rostered and selected via a preprogrammed selection algorithm to select zero to two individuals for the interview, depending on the composition of the household. Weighted response rates for the 2010–2014 NSDUHs ranged from 71.2 to 74.7%. Data for these analyses were restricted to about 107 300 adolescents aged 12–17 not sampled from group quarters (<0.25% of the sample annually), and exclude street- and shelter-dwelling homeless adolescents to focus on transience without homelessness confounding the results.

### Procedure

NSDUH interviews are conducted in the respondent's residence using computer-assisted interviewing (audio computer-assisted self-interviewing for sensitive topics). Verbal parental consent and adolescent assent for participation were obtained from all respondents. Data collection was completed in compliance with the International Code of Medical Ethics of the World Medical Association (Declaration of Helsinki) and the RTI International Institutional Review Board.

#### Measures

**Fig. 1.**
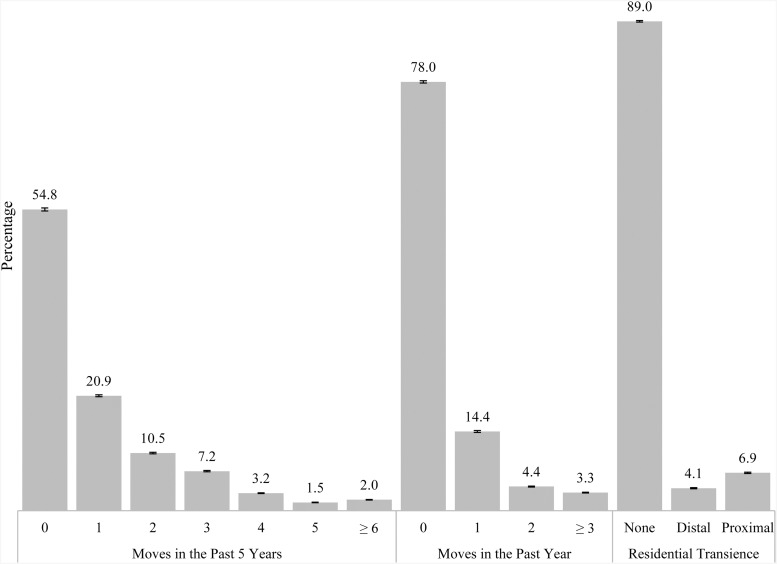
Number of moves and residential transience in the past 5 years among US adolescents aged 12–17: annual average percentages and standard errors. Source: SAMHSA, Center for Behavioral Health Statistics and Quality, National Surveys on Drug Use and Health, 2010–2014.

*Exposure*: Self-reported patterns of residential transience were assessed by asking how many times respondents moved in the past 5 years (0, 1, 2, 3, 4, 5 or ⩾6), then in the past year (0, 1, 2 or ⩾3). Prior research has not used a consistent definition of transience, for example, ⩾3 moves by age 7 (Gilman *et al*., 2003), number of moves by age 18 (Qin *et al*., 2009) or ⩾3 moves in the past year (Glasheen and Forman-Hoffman, 2015*a*, 2015*b*). The definition used in this study was determined with several considerations. First, the right-censored data (i.e. the data are capped at ⩾6 and ⩾3 moves) and wanting to examine distal *v.* recent transience precluded using a continuous number of moves. Second, prior research using NSDUH data defined past year transience as ⩾3 moves and we were interested in whether associations would be seen using a lower threshold. Third, transience is theoretically a high level of mobility, therefore we conducted an analysis examining the distribution of the number of past 5 year and past year moves in the population to set a threshold using <10% of the population as an indicator for ‘high level’ of mobility. Results of these analyses (Fig. 1) led us to define transience as recent transience (⩾2 moves in the past year, <8% of adolescents moved this frequently), distal transience (⩾4 moves in the past 5 years (<7% of adolescents moved this frequently) and no recent transience) and no transience (no recent or distal transience). Due to the small number of adolescents experiencing both recent and distal transience, they were combined with the recent transience group.

The two outcomes of interest were: past year MDE and past year mental health treatment. Past year MDE was defined per criteria from the fourth edition of the Diagnostic and Statistical Manual of Mental Disorders (DSM-IV). MDE was assessed among adolescents in NSDUH using a series of questions that ask about the nine DSM-IV criteria for MDE (LeBaron and McHenry, 2013). Adolescent questions were based on those used in the National Comorbidity Survey Replication Adolescent Supplement (NCS-A) (Kessler *et al*., 2009), with slight modifications based on cognitive interview findings (Center for Behavioral Health Statistics and Quality, 2015*a*). In the NCS-A clinical reappraisal study, MDE had a sensitivity of 80.4, a specificity of 95.3 and an area under the curve of 0.87. In NSDUH, test-retest reliability for past year MDE is good with *κ* = 0.72 (Substance Abuse and Mental Health Services Administration, 2010). In this sample, 9.5% (*n* = ~9900) of respondents met criteria for past year MDE.

Past year mental health treatment was assessed in two NSDUH sections. First, mental health treatment was assessed by asking all adolescents about treatment and counselling for any problems with behaviours or emotions that were not caused by alcohol or drugs, including treatment received in hospital inpatient settings; residential treatment centres; foster care/therapeutic foster care; partial day hospital/day treatment programmes; mental health clinics or centres; from a private therapist, psychologist, psychiatrist, social worker or counsellor; from an in-home therapist, counsellor or family preservation worker; from a paediatrician or other family doctor; and from a school social worker, psychologist or counsellor. Among adolescents, 22.1% had received any past year mental health treatment (*n* = ~24 200). The specific reason for this treatment was not assessed. Another question assessing past year treatment was asked as part of the MDE module. This question focused solely on treatment for MDE among adolescents with MDE. An estimated 38.6% of adolescents with past year MDE reported mental health treatment for depression (*n* = ~4000).

Other covariates were selected *a priori* and included in adjusted models based on past research into transience, depression and treatment, and were chosen for their potential to confound the relationship between transience and MDE or treatment. Covariates included gender (Davey-Rothwell *et al*., 2008), age (Mok *et al*., 2016), race/ethnicity (Atdjian and Vega, 2005; Glasheen and Forman-Hoffman, 2015*b*), number of parents (including step, foster and adopted parents) (Gilman *et al*., 2003; Crowder and Teachman, 2004), metropolitan area (Gilman *et al*., 2003), poverty status (Clark, 2010), health insurance (Jelleyman and Spencer, 2008; Ma *et al*., 2008), past year alcohol use and DSM-IV-defined alcohol use disorder (DeWit, 1998; Dong *et al*., 2005; Jelleyman and Spencer, 2008), past year drug use and DSM-IV-defined drug use disorder (DeWit, 1998; Jelleyman and Spencer, 2008) and past month tobacco use and tobacco dependence (Dong *et al*., 2005).

### Data analyses

Analyses were conducted using SUDAAN® to account for NSDUH's complex sample design (RTI International, 2012). Statistical tests were two tailed and tested at *α* = 0.05. Prevalence estimates represent weighted annual averages (i.e. the weighted average prevalence of the estimate across the data years). First, descriptive analyses were conducted, examining the annual average weighted prevalence of past 5-year and past year moves and distal and recent transience among all adolescents. Additionally, tests for trend were conducted to examine the linear relationship between the number of past 5-year moves and the odds of past year MDE.

Second, prevalence estimates of selected covariates by transience status were calculated. Overall statistical differences in characteristics by transience status were tested using Shah's Wald *F*-tests. Post hoc comparisons of proportions across each group were conducted using *t*-tests (rather than *χ*^2^), which better reflect the test statistic in complex survey data (Aldworth *et al*., 2012).

Third, three logistic regression models examined the odds of past year MDE and mental health treatment associated with transience status, controlling for potential confounders. One model examined the association of transience with MDE in adolescents and two models examined the odds of past year mental health treatment (any past year mental health service use and depression-specific mental health service use) among adolescents with MDE. Both outcomes were examined because it is not uncommon for those receiving mental health treatment to not know the specific symptoms/disorder they are being treated for at any given time, particularly if the adolescent present with multiple problem types (e.g. anxiety co-occurring with depression). Therefore, any mental health treatment will include treatment that may address depression symptoms while not being recognised as depression-specific treatment. Results were similar for any mental health treatment and treatment for depression, therefore results for treatment of depression are presented in the online Supplementary materials. Tests for collinearity and multicollinearity were completed for each model (Liao and Valliant, 2012).

Finally, a sensitivity analysis was conducted. Transience was assessed using two separate questions. Respondents who reported past 5-year moves were asked about the number of past year moves without data quality constraints. This led to some adolescents reporting more moves in the past year than they reported in the past 5 years. Additionally, some respondents answered ‘don't know’ to the past 5-year question but answered the past year question and *vice versa*. A sensitivity analysis was conducted to address the impact of reporting error. Results indicated almost no differences between the models; therefore, we present model results from only the listwise deletion models (specific model results are provided in the online Supplementary Materials).

## Results

### Characteristics associated with residential transience

More than half (54.8%) of all adolescents aged 12–17 had not moved in the past 5 years, and 78.0% had not moved in the past year (Fig. 1). About 11.0% of adolescents reported some residential transience in the past 5 years; 4.1% (weighted *N* = ~971 000) experienced distal transience, and 6.9% (weighted *N* = ~1 658 000) experienced recent transience.

Adolescents with distal or recent transience differed for all covariates examined except for gender (*p* =  0.640; Table 1). Adolescents with transience tended to be younger than adolescents without transience. Adolescents with transience were more likely to be non-Hispanic black, non-Hispanic of more than one race and Hispanic compared with adolescents without any transience in the past 5 years. Adolescents without transience were more likely to be non-Hispanic white or non-Hispanic Asian, compared with adolescents with transience. Adolescents without transience were more likely to report living in a large metropolitan area than those with transience, but they were less likely to report living in a small metropolitan area compared with those with transience. Adolescents experiencing transience in the past 5 years were more likely to report zero or one parent than adolescents without transience, whereas adolescents reporting no transience were more likely to report two or more parents. Adolescents with transience in the past 5 years were more likely to have a family income below 200% of the federal poverty level compared with those without transience. Adolescents with transience were also less likely to have health insurance compared with those without transience. Substance use was more likely among adolescents who had transience compared with those without transience.

**Table 1.** Characteristics of adolescents aged 12–17, by past year residential transience status, annual averages: weighted *N* (in thousands), percentages and standard errors

Characteristic	No transience (*N* = 21 077.8)		Distal transience^a^ (*N* = 971.0)		Recent transience (*N* = 1658.0)^b^	
	Percentage	Standard error	Percentage	Standard error	Percentage	Standard error
Gender						
Male	51.1	(0.22)	50.3	(1.03)	50.6	(0.78)
Female	48.9	(0.22)	49.7	(1.03)	49.4	(0.78)
Age						
12–13	31.1	(0.20)^c^	37.6	(0.95)^c^^,^^d^	32.8	(0.75)^d^
14–15	33.9	(0.20)	34.7	(0.96)	34.1	(0.75)
16–17	35.0	(0.20)^c^	27.7	(0.90)^c^^,^^d^	33.1	(0.72)^d^
Race/ethnicity						
NH White	57.7	(0.35)^c^	45.6	(1.05)^c^^,^^d^	41.6	(0.84)^d^
NH Black	12.6	(0.23)^c^	19.6	(0.87)^c^^,^^d^	25.8	(0.77)^d^
NH American Indian/Alaska Native	0.6	(0.04)	0.9	(0.16)	0.8	(0.12)
NH Native Hawaiian/other Pacific Islander	0.3	(0.03)	0.4	(0.12)	0.6	(0.13)
NH Asian	5.1	(0.16)^c^	2.8	(0.39)^d^	2.5	(0.28)^d^
NH multiple races	2.7	(0.07)^c^	4.2	(0.38)^d^	3.8	(0.29)^d^
Hispanic	21.0	(0.28)^c^	26.6	(1.00)^d^	25.0	(0.81)^d^
Metropolitan area						
Large	56.5	(0.37)^c^	53.6	(1.09)^d^	51.7	(0.90)^d^
Small	29.7	(0.37)^c^	31.4	(0.97)^c^	34.1	(0.87)^d^
Non-metropolitan	13.8	(0.24)	15.0	(0.76)	14.1	(0.58)
Number of parents						
None or 1	27.8	(0.24)^c^	48.9	(1.06)^c^^,^^d^	54.4	(0.86)^d^
2 or more	72.2	(0.24)^c^	51.1	(1.06)^c^^,^^d^	45.6	(0.86)^d^
Poverty status						
<100% FPL	19.0	(0.25)^c^	36.4	(1.03)^c^^,^^d^	42.7	(0.87)^d^
100–199% FPL	21.5	(0.22)^c^	27.9	(0.95)^d^	26.2	(0.71)^d^
⩾200% FPL	59.5	(0.32)^c^	35.7	(1.04)^c^^,^^d^	31.1	(0.80)^d^
Health insurance						
Yes	93.9	(0.13)^c^	91.2	(0.63)^c^^,^^d^	88.9	(0.56)^d^
No	6.1	(0.13)^c^	8.8	(0.63)^c^^,^^d^	11.1	(0.56)^d^
Tobacco dependence						
No PM use	91.7	(0.12)^c^	88.1	(0.64)^d^	86.6	(0.53)^d^
PM use but no dependence	6.3	(0.11)^c^	8.7	(0.56)^d^	8.2	(0.45)^d^
PM dependence	2.0	(0.06)^c^	3.2	(0.32)^c^^,^^d^	5.2	(0.33)^d^
Alcohol use disorder (AUD)						
No PY use	73.9	(0.19)^c^	71.0	(0.92)^d^	70.1	(0.74)^d^
PY use but no AUD	22.8	(0.18)^c^	25.0	(0.89)^d^	24.9	(0.68)^d^
PY AUD	3.3	(0.08)^c^	4.1	(0.42)	5.0	(0.33)^d^
Illicit drug use disorder (DUD)						
No PY use	82.7	(0.17)^c^	76.4	(0.83)^c^^,^^d^	74.3	(0.69)^d^
PY use but no DUD	13.6	(0.15)^c^	17.2	(0.73)^d^	18.9	(0.62)^d^
PY DUD	3.7	(0.09)^c^	6.5	(0.49)^d^	6.8	(0.37)^d^

FPL, federal poverty level; NH, non-Hispanic; PM, past month; PY, past year.

*Source*: SAMHSA, Center for Behavioral Health Statistics and Quality, National Surveys on Drug Use and Health, 2010–2014.

a Distal transience was defined as ⩾4 moves in the past 5 years, but no recent transience.

b Recent transience was defined as ⩾2 moves in the past year.

c Statistical comparison with recent transience is significant at *p* < 0.05.

d Statistical comparison with no residential transience is significant at *p* < 0.05.

### Residential transience and past year MDE

**Fig. 2.**
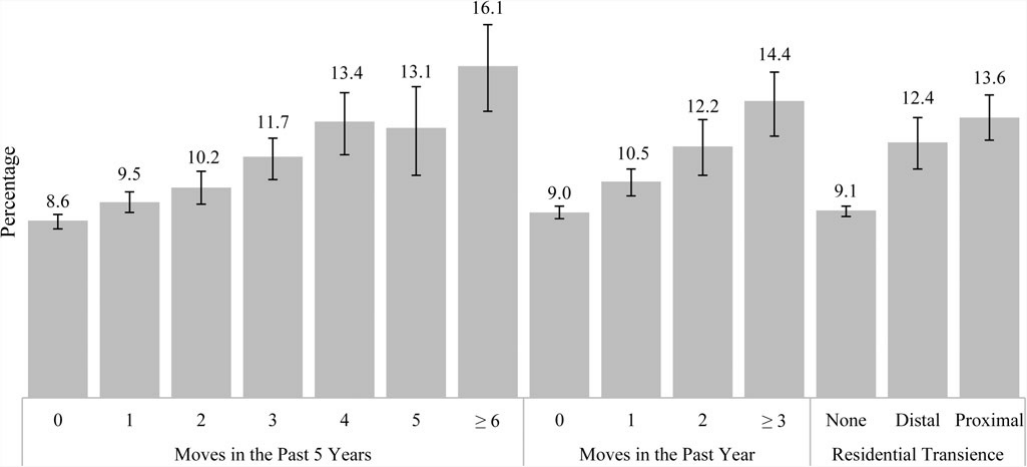
Prevalence of past year major depressive episode among US adolescents aged 12–17: annual average percentages and 95% confidence intervals. Source: SAMHSA, Center for Behavioral Health Statistics and Quality, National Surveys on Drug Use and Health, 2010–2014.

Past year MDE prevalence increased with the number of moves in the past 5 and past year (tests for trend *p* < 0.001; Fig. 2). MDE prevalence was greater among adolescents with distal or recent transience, compared with those without transience (12.4 *v*. 9.1, *p* < 0.001 and 13.6 *v*. 9.1, *p* < 0.001, respectively). There was no difference in the prevalence of MDE among those who had distal transience compared with those who had recent transience (12.4 *v*. 13.6, *p* = 0.163). Adjusting for demographics, socioeconomic status, substance use and mental health treatment, transience remained significantly associated with past year MDE. The adjusted odds of MDE were 25% greater among those with distal transience (adjusted odds ratio (AOR) = 1.25, 95% confidence interval (CI) = 1.09–1.44) and 31% greater among those with recent transience (AOR = 1.31, 95% CI = 1.17–1.46) compared with those without transience (Table 2).

**Table 2.** Past year MDE and mental health treatment among adolescents aged 12–17: annual averages, adjusted odds ratios (AOR) and 95% confidence intervals (95% CI)

Variable	Past year MDE among adolescents		Any past year mental health treatment among adolescents with MDE	
	AOR	95% CI	AOR	95% CI
Residential transience				
None	1.00		1.00	
Distal (past 5 years but not past year)	1.25	(1.09–1.44)	1.18	(0.92–1.51)
Recent (past year)	1.31	(1.17–1.46)	1.40	(1.15–1.70)
Gender				
Male	0.33	(0.31–0.35)	0.72	(0.64–0.82)
Female	1.00		1.00	
Age				
12–13	0.50	(0.46–0.55)	1.58	(1.33–1.87)
14–15	0.89	(0.83–0.95)	1.40	(1.24–1.59)
16–17	1.00		1.00	
Race/ethnicity				
NH White	1.00		1.00	
NH Black	0.75	(0.67–0.82)	0.70	(0.58–0.84)
NH American Indian/Alaska Native	0.63	(0.45–0.87)	1.11	(0.61–1.99)
NH Native Hawaiian/other Pacific Islander	0.82	(0.44–1.53)	0.49	(0.14–1.71)
NH Asian	1.03	(0.87–1.22)	0.71	(0.51–1.00)
NH multiple races	1.05	(0.89–1.23)	1.07	(0.81–1.41)
Hispanic	1.05	(0.97–1.15)	0.78	(0.67–0.90)
Metropolitan area				
Large	0.99	(0.91–1.08)	1.15	(0.98–1.34)
Small	1.00	(0.92–1.08)	1.14	(0.97–1.33)
Non-metropolitan	1.00		1.00	
Poverty status				
<100% FPL	0.96	(0.88–1.05)	0.89	(0.76–1.04)
100–199% FPL	1.08	(1.00–1.17)	0.86	(0.75–1.00)
⩾200% FPL	1.00		1.00	
Health insurance				
Yes	1.03	(0.91–1.16)	1.51	(1.19–1.92)
No	1.00		1.00	
Tobacco dependence				
No PM use	1.00		1.00	
PM use but no dependence	0.91	(0.82–1.01)	1.06	(0.88–1.27)
PM dependence	0.61	(0.52–0.73)	1.46	(1.10–1.94)
Alcohol use disorder (AUD)				
No PY use	1.00		1.00	
PY use but no AUD	1.41	(1.30–1.52)	0.91	(0.80–1.04)
PY AUD	1.95	(1.70–2.25)	1.16	(0.92–1.46)
Illicit drug use disorder (DUD)				
No PY use	1.00		1.00	
PY use but no DUD	1.53	(1.40–1.68)	1.22	(1.06–1.41)
PY DUD	2.39	(2.09–2.74)	1.22	(0.99–1.50)
Number of parents				
None or 1	1.05	(0.98–1.13)	1.33	(1.18–1.50)
2 or more	1.00		1.00	
Any past year mental health treatment	3.70	(3.48–3.93)	N/A	

FPL, federal poverty level; MDE, major depressive episode; N/A, not applicable; NH, non-Hispanic; PM, past month; PY, past year.

*Source*: SAMHSA, Center for Behavioral Health Statistics and Quality, National Surveys on Drug Use and Health, 2010–2014.

### Residential transience and past year mental health treatment

**Fig. 3.**
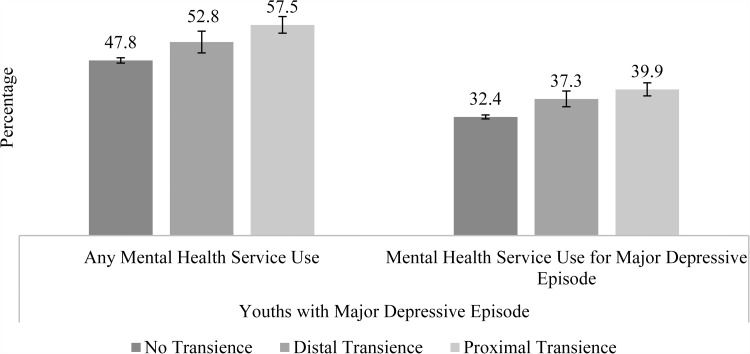
Prevalence of past year mental health treatment among US adolescents aged 12–17 who had a past year major depressive episode: annual average percentages and 95% confidence intervals. Source: SAMHSA, Center for Behavioral Health Statistics and Quality, National Surveys on Drug Use and Health, 2010–2014.

Figure 3 shows the prevalence of mental health treatment among adolescents with MDE by transience status. The prevalence of treatment was higher among adolescents with recent transience compared with those without transience (57.5 *v*. 47.8%, *p* < 0.001). In models adjusting for demographics, socioeconomic status and substance use, the adjusted odds of treatment remained higher among those with recent transience compared with those without (AOR = 1.40, 95% CI = 1.15–1.70), but there was no significant difference in the odds of treatment among adolescents with distal transience and those without transience (AOR = 1.18, 95% CI = 0.92–1.51).

## Discussion

Parents, school officials and health care providers should be aware that residential mobility in the past 5 years may indicate increased odds of depression among adolescents and that this remained elevated among adolescents with transience in the past 5 years even when the transience occurred more than 1 year prior. Moreover, the relationship between mobility and MDE followed a clear dose–response pattern, with the highest prevalence of MDE occurring among adolescents with the largest number of moves. One in six adolescents who had moved six or more times in the past 5 years and one in seven adolescents who had moved three or more times in the past year reported past year MDE. These findings are consistent with prior literature that demonstrated an increased risk of depression and other negative health outcomes among adolescents with frequent mobility (Gilman *et al*., 2003; Jelleyman and Spencer, 2008; Susukida *et al*., 2015). Moreover, it suggests that transience may not be a discrete construct and that number of moves has a cumulative rather than threshold effect. Examinations of this dose–response pattern in tandem with more information on other life stressors are needed to evaluate whether residential relocation behaves like a generic stressor or has a particular association with MDE. This may also be explored by examining differences in the types of moves (e.g. same neighbourhood *v*. different neighbourhood *v*. different state), which could help determine whether the relocation itself is particularly stressful or whether certain associated characteristics such as changing schools is a more important component. Future studies may want to examine this further by looking at the association between depression and transience using a continuous variable for the number of moves (when data permit).

We constructed the transience analyses to examine whether there was a stronger association with recent *v.* distal only mobility. Although the prevalence of MDE was higher among those with recent transience compared with those with distal transience, this difference was not significant, which was contrary to our hypothesis. Several factors may be responsible for this observation. First, MDE onset may be closer to the timing of the mobility but has persisted even among those who have not had transience in the past year. Although the minimum duration of MDE is 2 weeks, depressive episodes in children average from 7 to 9 months but can last years, and recurrent episodes are not uncommon (Cicchetti and Toth, 1998). NSDUH does not collect MDE age-of-onset data and is a cross-sectional measure without external verification; therefore, future research is needed to examine the timing of the onset of MDE and residential mobility, preferably using a longitudinal design that better captures order of onset and incidence data. Second, combining individuals with both recent and distal transience into the recent transience group may have obscured the difference in MDE prevalence for recent *v.* distal only transience. However, despite combining 11 years of data the small sample sizes for adolescents experiencing both recent and distal transience were too small for analysis. Additional research would benefit from examining those with longstanding (e.g. both recent and distal) transience as they may be at an even more substantial risk for mental health problems. Furthermore, NSDUH only captures mobility in the past 5 years; future research is needed to examine even more distal moves so that the cumulative effects of transience can be explored. More examination is needed for MDE and across other outcomes because the associations between transience and other mental health outcomes may not be similarly linear. A third reason for not identifying a difference in MDE among those with recent *v.* distal transience may be an unmeasured confounder responsible for the apparent association between transience and MDE. Although our analyses controlled for several housing-related variables, including demographic and socioeconomic indicators (e.g. age, race/ethnicity, poverty level, number of parents), this secondary data analysis was limited to available covariates and could not control for all potentially related covariates (e.g. parent's mental health, housing characteristics, recent stressful events or social support).

Another important consideration in evaluating the association between transience and MDE is the timing of moves in relationship to developmental milestones such as puberty. Puberty is a time of significant physiological maturation and a window in which the incidence of disorders such as MDE begins to dramatically increase, and gender differences in the rates of these disorders start to become apparent. The average start of puberty for girls is between 10 and 12 years of age and boys between 11 and 12; moreover, the individual variation can range by 4–5 years (Parent *et al*., 2003). Unfortunately, NSDUH does not collect data on pubertal stage and collects data only from people aged 12 or older. Therefore, the data cannot be used to differentiate between those who are going through puberty and those who have finished puberty. Whether there are important developmental periods during which moving can be particularly disruptive and contribute specifically to mental illness onset is yet to be determined but an important area for future, longitudinal research. Additionally, children rarely have a say in their housing situation, and housing relocations are usually a result of factors beyond their control, such as poverty, parental mental illness, parental employment changes, divorce or separation. Future research that uses data from multiple domains would make a strong contribution towards better understanding the impact of transience on adolescents’ mental health from a more holistic perspective that examines developmental stage and family dynamics.

Contrary to our hypotheses and prior research suggesting mobility may be a barrier to care (Jelleyman and Spencer, 2008), transience was not associated with lower rates of mental health treatment among adolescents with MDE. Adolescents with MDE and past year transience were more likely to report any treatment compared with those without transience in the past 5 years. However, distal transience was not associated with an increase in past year treatment. This may be due to the cross-sectional nature of the data and the concurrent measure of recent transience with past year treatment, whereas distal transience was outside of the assessment period for treatment. Parents and other involved adults (e.g. school administrators, teachers) may also be more alert to the possibility of adjustment challenges and behavioural health problems among children who recently moved, but this awareness fades further from the move. Our findings indicated that adolescents with distal transience only were still at increased risk of MDE of similar magnitude to the risk of MDE among adolescents with more recent transience. This suggests the importance of having parents and other involved adults maintain awareness of the potential for MDE, even after the residential situation has stabilised.

Despite the findings that recent transience was associated with higher rates of treatment, it is premature to conclude that transience does not act as a barrier to mental health care. Among adolescents, NSDUH collects data on only MDE and substance use disorders; therefore, other unmeasured disorders may affect these results. Notably, all three substance use problems (tobacco, alcohol and illicit drugs) were associated with transience. Adolescents with transience may have more severe symptoms or comorbidity, thereby prompting a higher frequency of treatment. Future research is needed for a nuanced evaluation that controls for the severity of mental health symptoms. Additionally, our definition of no transience (<4 moves in 5 years and <2 moves in the past year) is conservative. Under this definition, adolescents who moved every 16 months would have been classified as having no transience. Although this reflects the concept that transience is an extreme event, it may obscure significant associations due to the high threshold. Regardless, adolescents who have moved frequently in the past 5 years are at higher risk of MDE and parents, school officials and health care providers should be aware that this increased risk does not appear to be reduced after a year of residential stability. Therefore, depression screening may be important to identifying depressed adolescents and referring them to treatment (Forman-Hoffman *et al*., 2016). Housing stability programmes may also be an important avenue for identifying adolescents at risk of MDE, as they may be able to inform parents to be more aware of this risk in their adolescents and/or provide screening for depression depending on their programme capabilities.

## Supplementary material

For supplementary material accompanying this paper visit https://doi.org/10.1017/S2045796018000823. click here to view supplementary material

## Availability of data and materials

Analyses were conducted using the restricted use files of the 2010–2014 NSDUHs. Restricted use files are not available for public use to ensure respondent confidentiality. However, NSDUH public use files are available from the Substance Abuse and Mental Health Data Archive at https://www.datafiles.samhsa.gov/.
