# Supplementary material for: Residential transience among US adolescents: association with depression and mental health treatment
Source: Epidemiol Psychiatr Sci. 2019 Jan 15;28(6):682–91. doi: 10.1017/S2045796018000823 (PMC6998884; doi:10.1017/S2045796018000823)
Supplement: Supplementary file 1 [file S2045796018000823sup001.docx]

Residential Transience among United States Adolescents: Association with Depression and Mental Health Treatment

SUPPLEMENTAL MATERIAL

Authors: C. Glasheen,^[[1]](#footnote-1)^ V. Forman-Hoffman,^1^ S. Hedden,^2^ T. Ridenour,^1^ J. Wang,^3^ J. Porter^3^

^1^ Behavioral Health Research Division, RTI International

^2^Center for Behavioral Statistics and Quality, Substance Abuse and Mental Health Services Administration

^3^Division for Statistical & Data Sciences, RTI International

# SUPPLEMENTAL MATERIAL

## S1. Sensitivity Analysis of Recoding Incongruent Past Year and Past 5 Year Moves

Transience was assessed using two separate questions. Respondents who reported past 5-year moves were asked about the number of past year moves without data quality constraints. This led to some adolescents reporting more moves in the past year than they reported in the past 5 years. Additionally, some respondents answered "don't know" to the past 5-year question but answered the past year question and vice versa. Analyses were conducted to see if missing or inconsistent mobility data was associated with the three outcomes of interest (past year Major Depressive Episodes (MDE), any past year mental health treatment among adolescents with MDE, and past year depression mental health treatment among those with MDE.

Missing/inconsistent data status was significantly associated with MDE and treatment (p<.05). However, less than 1 percent of the sample had missing or inconsistent data. Therefore, a sensitivity analysis was conducted to address the impact of reporting error. The primary analysis set all respondents who had incomplete or inconsistent responses to missing, which is what is presented in the main paper. Two additional sets of frequency analyses were conducted for the MDE outcome (Table S1) and Depression Treatment among those with MDE (Table S2). One analysis set the value of past 5 year moves equal to the number of past year moves when missing or inconsistent, the other set the value of past year moves to equal past 5 year moves when missing or inconsistent. Changes in the frequency results for the three sets of models were negligible.

**Table S1. Youth Number of Moves and Residential Transience by Past Year Major Depressive Episodes, 2010-2014 Annual Averages: Percentages, Standard Errors of Percentages, and 95% Confidence Intervals. National Survey on Drug Use and Health**

|  | **No MDE** | | **MDE** | |
| --- | --- | --- | --- | --- |
|  | **% (S.E.)** | **95% CI** | **% (S.E.)** | **95% CI** |
| **Transience (Listwise Deletion)** | | | | |
| None | 89.5 (0.15) | 89.2-89.8 | 85.0 (0.49) | 84.0-85.9 |
| Past 5 Years but not Past Year | 3.9 (0.09) | 3.8-4.1 | 5.2 (0.28) | 4.7-5.8 |
| Past Year | 6.6 (0.12) | 6.4-6.8 | 9.8 (0.42) | 9.0-10.6 |
| **Transience (Sensitivity 1: past 5-year moves set to past year)** | | | | |
| None | 88.9 (0.16) | 88.6-89.2 | 84.4 (0.50) | 83.4-85.4 |
| Past 5 Years but not Past Year | 3.9 (0.09) | 3.7-4.1 | 5.2 (0.28) | 4.7-5.8 |
| Past Year | 7.2 (0.13) | 7.0-7.5 | 10.4 (0.44) | 9.5-11.3 |
| **Transience (Sensitivity 2: past year moves set to past 5-year)** | | | | |
| None | 89.2 (0.16) | 88.9-89.5 | 84.7 (0.49) | 83.7-85.6 |
| Past 5 Years but not Past Year | 3.9 (0.09) | 3.7-4.1 | 5.2 (0.28) | 4.7-5.8 |
| Past Year | 6.9 (0.12) | 6.7-7.1 | 10.1 (0.43) | 9.3-11.0 |

**Table S2. Estimates of Number of Moves and Residential Transience among Youth with Major Depressive Episode (MDE) by Transience Status, 2010-2014 Annual Averages: Percentages, Standard Errors of Percentages, and 95% Confidence Interval (CI)**

|  | **No Service Use for Depression** | | **Service Use for Depression** | |
| --- | --- | --- | --- | --- |
|  | **% (S.E.)** | **95% CI** | **% (S.E.)** | **95% CI** |
| **Transience (Listwise Deletion)** | | | | |
| None | 86.5 (0.60) | 85.2-87.6 | 82.6 (0.83) | 80.9-84.2 |
| Past 5 Years but not Past Year | 5.0 (0.37) | 4.3-5.7 | 5.7 (0.47) | 4.8-6.7 |
| Past Year | 8.6 (0.51) | 7.6-9.6 | 11.7 (0.72) | 10.4-13.2 |
| **Transience (Sensitivity 1: past 5-year moves set to past year)** | | | | |
| None | 85.9 (0.61) | 84.6-87.1 | 82.2 (0.83) | 80.5-83.7 |
| Past 5 Years but not Past Year | 4.9 (0.36) | 4.2-5.7 | 5.6 (0.47) | 4.8-6.6 |
| Past Year | 9.2 (0.52) | 8.2-10.3 | 12.2 (0.73) | 10.8-13.7 |
| **Transience (Sensitivity 2: past year moves set to past 5-year)** | | | | |
| None | 86.2 (0.61) | 85.0-87.3 | 82.3 (0.83) | 80.6-83.9 |
| Past 5 Years but not Past Year | 4.9 (0.36) | 4.2-5.7 | 5.6 (0.47) | 4.8-6.6 |
| Past Year | 8.9 (0.52) | 7.9-10.0 | 12.1 (0.73) | 10.7-13.6 |

## S2. Depression Treatment among Adolescents with Past Year Major Depressive Episode(s) (MDE)

Associations between residential transience and mental health treatment were similar for models examining any past year mental health treatment and treatment for depression (Table S3) Adolescents with recent transience were more likely to report treatment for MDE in the past year than those with no transience in the past 5 years (AOR = 1.43, 95% = 1.17-1.74), but there was no significant difference in the likelihood of treatment for MDE for those with distal transience compared with adolescents with MDE with no transience (AOR = 1.23, 95% CI = 0.96-1.57).

Table S3 Past Year MDE and Mental Health Treatment among Adolescents Aged 12 to 17: Annual Averages, Adjusted Odds Ratios (AOR), and 95% Confidence Intervals (95% CI)

| Variable | Any Past Year Mental Health Treatment among Adolescents with MDE | | Past Year Mental Health Treatment for Depression among Adolescents with MDE | |
| --- | --- | --- | --- | --- |
|  | AOR | 95% CI | AOR | 95% CI |
|  | | | | |
| None | 1.00 |  | 1.00 |  |
| Distal (Past 5 Years but Not Past Year) | 1.18 | (0.92-1.51) | 1.23 | (0.96-1.57) |
| Recent (Past Year) | 1.40 | (1.15-1.70) | 1.43 | (1.17-1.74) |
| Gender | | | | |
| Male | 0.72 | (0.64-0.82) | 0.69 | (0.61-0.79) |
| Female | 1.00 |  | 1.00 |  |
| Age | | | | |
| 12-13 | 1.58 | (1.33-1.87) | 0.80 | (0.68-0.95) |
| 14-15 | 1.40 | (1.24-1.59) | 0.88 | (0.78-1.00) |
| 16-17 | 1.00 |  | 1.00 |  |
| Race/Ethnicity | | | | |
| NH White | 1.00 |  | 1.00 |  |
| NH Black | 0.70 | (0.58-0.84) | 0.65 | (0.54-0.78) |
| NH American Indian/Alaska Native | 1.11 | (0.61-1.99) | 1.03 | (0.54-1.96) |
| NH Native Hawaiian/Other Pacific Islander | 0.49 | (0.14-1.71) | 0.71 |  |
| NH Asian | 0.71 | (0.51-1.00) | 0.43 | (0.29-0.64) |
| NH Multiple Races | 1.07 | (0.81-1.41) | 0.96 | (0.72-1.28) |
| Hispanic | 0.78 | (0.67-0.90) | 0.70 | (0.60-0.81) |
| Metropolitan Area | | | | |
| Large | 1.15 | (0.98-1.34) | 1.19 | (1.01-1.39) |
| Small | 1.14 | (0.97-1.33) | 1.09 | (0.92-1.29) |
| Nonmetropolitan | 1.00 |  | 1.00 |  |
| Poverty Status | | | | |
| < 100% FPL | 0.89 | (0.76-1.04) | 0.90 | (0.77-1.05) |
| 100-199% FPL | 0.86 | (0.75-1.00) | 1.03 | (0.89-1.19) |
| ≥ 200% FPL | 1.00 |  | 1.00 |  |
| Health Insurance | | | | |
| Yes | 1.51 | (1.19-1.92) | 1.48 |  |
| No | 1.00 |  | 1.00 |  |
|  | | | | |
| No PM Use | 1.00 |  | 1.00 |  |
| PM Use but No Dependence | 1.06 | (0.88-1.27) | 1.20 | (0.99-1.45) |
| PM Dependence | 1.46 | (1.10-1.94) | 1.32 | (1.01-1.73) |
|  | | | | |
| No PY Use | 1.00 |  | 1.00 |  |
| PY Use but No AUD | 0.91 | (0.80-1.04) | 0.82 | (0.72-0.94) |
| PY AUD | 1.16 | (0.92-1.46) | 0.98 | (0.78-1.23) |
|  | | | | |
| No PY Use | 1.00 |  | 1.00 |  |
| PY Use but No DUD | 1.22 | (1.06-1.41) | 1.23 | (1.06-1.42) |
| PY DUD | 1.22 | (0.99-1.50) | 1.41 | (1.15-1.74) |
| Number of Parents | | | | |
| None or 1 | 1.33 | (1.18-1.50) | 1.20 |  |
| 2 or more | 1.00 |  | 1.00 |  |

1. Corresponding author. Cristie Glasheen, RTI International, 3040 E. Cornwallis Road, Research Triangle Park, NC 27709 [↑](#footnote-ref-1)
